# Identification of a Drug Targeting an Intrinsically Disordered Protein Involved in Pancreatic Adenocarcinoma

**DOI:** 10.1038/srep39732

**Published:** 2017-01-05

**Authors:** José L. Neira, Jennifer Bintz, María Arruebo, Bruno Rizzuti, Thomas Bonacci, Sonia Vega, Angel Lanas, Adrián Velázquez-Campoy, Juan L. Iovanna, Olga Abián

**Affiliations:** 1 Instituto de Biología Molecular y Celular, Universidad Miguel Hernández, Edificio Torregaitán, Avda. del Ferrocarril s/n, 03202 Elche, Alicante, Spain; 2 Instituto de Biocomputación y Física de Sistemas Complejos (BIFI), Unidad Asociada IQFR-CSIC-BIFI, Universidad de Zaragoza, Edificio I+D, Mariano Esquillor s/n, 50018 Zaragoza, Spain; 3 Centre de Recherche en Cancérologie de Marseille (CRCM), INSERM U1068, CNRS UMR 7258, Aix-Marseille Université and Institut Paoli-Calmettes, Parc Scientifique et Technologique de Luminy, 163 Avenue de Luminy, 13288, Marseille, France; 4 Instituto Aragonés de Ciencias de la Salud (IACS), Av. San Juan Bosco 13, 50009 Zaragoza, Spain; 5 Instituto de Investigaciones Sanitarias (IIS) Aragón, Av. San Juan Bosco, 13, 50009 Zaragoza, Spain; 6 CNR-NANOTEC, Licryl-UOS Cosenza and CEMIF.Cal, Department of Physics, University of Calabria, Via P. Bucci, Cubo 31 C, 87036 Arcavacata di Rende, Cosenza, Italy; 7 Centro de Investigación Biomédica en Red en el Área Temática de Enfermedades Hepáticas y Digestivas (CIBERehd), Spain; 8 Servicio de Aparato Digestivo, Hospital Clínico Universitario “Lozano Blesa”, Av. San Juan Bosco, 15, 50009 Zaragoza, Spain; 9 Department of Medicine, University of Zaragoza, Perdro Cerbuna 12, 50009 Zaragoza, Spain; 10 Fundación ARAID, Diputación General de Aragón, C/María de Luna 11, Edificio CEEIARAGÓN, 50018 Zaragoza, Spain

## Abstract

Intrinsically disordered proteins (IDPs) are prevalent in eukaryotes, performing signaling and regulatory functions. Often associated with human diseases, they constitute drug-development targets. NUPR1 is a multifunctional IDP, over-expressed and involved in pancreatic ductal adenocarcinoma (PDAC) development. By screening 1120 FDA-approved compounds, fifteen candidates were selected, and their interactions with NUPR1 were characterized by experimental and simulation techniques. The protein remained disordered upon binding to all fifteen candidates. These compounds were tested in PDAC-derived cell-based assays, and all induced cell-growth arrest and senescence, reduced cell migration, and decreased chemoresistance, mimicking NUPR1-deficiency. The most effective compound completely arrested tumor development *in vivo* on xenografted PDAC-derived cells in mice. Besides reporting the discovery of a compound targeting an intact IDP and specifically active against PDAC, our study proves the possibility to target the ‘fuzzy’ interface of a protein that remains disordered upon binding to its natural biological partners or to selected drugs.

The discovery of new ligands binding to a biomolecule represents the first step in the development of therapeutic drugs^1^. For drugs based on small organic ligands, high-throughput screening is the most popular approach: large libraries of compounds are synthesized (or purchased), and each compound is assayed for the binding to the target, although in most cases further chemistry is required to improve specificity and binding affinity^2^. In the last years, much of the effort on drug-development has been focused in understanding protein-protein interactions (PPIs) as potential targets. It has been shown that the free-energy of PPIs, even displaying large binding interfaces, is determined by rather specific regions whose surfaces can be matched by small molecules: the so-called hot-spot regions^3^.

Intrinsically disordered proteins (IDPs) do not have stable secondary or tertiary structures in several regions, or throughout their whole sequence^4^,^5^,^6^, since they exist as an ensemble of rapidly inter-converting structures. Because of their plasticity IDPs act as hubs in interaction networks carrying out several functions in cell-signaling routes and regulation (“moonlighting”)^5^,^6^, thus they are very often involved in important diseases. IDPs are present in all kingdoms of life: in eukaryotic cells, more than 40% of the proteins possess disordered regions longer than 50 residues^6^. Thus, IDPs are recognized as potential drug targets^7^, although the current design strategies for drugs acting on well-folded proteins are not appropriate for IDPs, due to their highly dynamic nature and the absence of a well-defined structure. Therefore, drug-selection for targeting IDPs is challenging and poses high difficulties to our current knowledge about PPIs.

The *nuclear protein 1 (NUPR1*, also known as p8 or *COM1*) gene was first described as overexpressed in acinar cells of the pancreas during the acute phase of pancreatitis^8^. The corresponding NUPR1 protein is an IDP, which binds DNA and is a substrate for protein kinase A; phosphorylation seems to increase its content of structure and the phosphorylated species also binds DNA^9^. The exact function of NUPR1 is only partially determined, intervening with Kras^G12D^ in modulation of precancerous lesions^10^,^11^,^12^. In fact, *NUPR1* expression controls pancreatic cancer cell migration, invasion and adhesion, three processes required for metastasis through CDC42, which is a major regulator of cytoskeleton organization^11^,^13^; apoptosis by interacting with prothymosin α^14^; and chemo-resistance^15^. Moreover, NUPR1 depletion in pancreatic ductal adenocarcinoma (PDAC)-derived cells, by using genetic approaches, results in cell-cycle arrest and senescence induction^16^. NUPR1 has also a role in regulating autophagy^17^, and in DNA-damage response through binding to male-specific-lethal protein 1 (MSL1), a histone acetyl transferase-associated protein^18^,^19^. NUPR1 is, therefore, a multifunctional protein involved in PDAC development and progression and a candidate to be pharmacologically targeted.

Here, we describe a comprehensive approach for drug-selection against NUPR1. We applied a methodology based on the synergy of biophysical, computational and biological methods to identify a drug against NUPR1. We started by screening 1120 Food and Drug Administration (FDA)-approved drugs (Prestwick Chemical Library) searching for compounds capable of binding to NUPR1 using fluorescence thermal-denaturation. Those triggering the largest changes in the thermal-denaturation profile (15 compounds) were examined by isothermal titration calorimetry (ITC) and nuclear magnetic resonance (NMR) to determine their binding affinity and the interacting region in NUPR1. In parallel and blindly, we carried out *in silico* studies to obtain models of the structures of the complexes between NUPR1 and the fifteen compounds. The models of the complexes showed that the selected compounds bind to a restricted number of residues in NUPR1, whose intensities in the NMR spectra changed slightly in the presence of the corresponding compound. The compounds were also assayed in PDAC-derived cell-based experiments to test whether they inhibited the interaction between MSL1 and NUPR1 *in vivo*; this interaction is critical during DNA-repair processes. All of the compounds induced cell-growth arrest, senescence, reduction in cell migration, and inhibited the interaction between the two proteins. Compound-15, the most effective one, was finally tested *in vivo* and completely arrested PDAC development in mice with tumor induced by xenografting PDAC-derived cells.

## Results

### Experimental screening: Identification of compounds interacting with NUPR1

NUPR1 is mostly unfolded, as shown by its CD and NMR spectra in isolation^9^,^19^. However, there is evidence of local, labile structure that might be stabilized by interacting ligands^20^. This protein-ligand interaction may promote some limited structural rearrangements, resulting in a different thermal denaturation pattern compared to the unliganded protein, which can be monitored by fluorescence using 8-anilino-1-naphthalene sulfonic acid (ANS) as an extrinsic probe. Therefore, ligand-induced stabilization against thermal denaturation can be employed for identifying potential NUPR1 interacting compounds. It is well-known that ANS interacts with hydrophobic patches in proteins; interestingly, although in general ANS exhibits an increase in fluorescence intensity upon protein unfolding, in some proteins and protein complexes there is a decrease in fluorescence intensity upon protein unfolding or complex dissociation, depending on the change in hydrophobicity of the solvent-exposed surface area.

**Figure 1:**
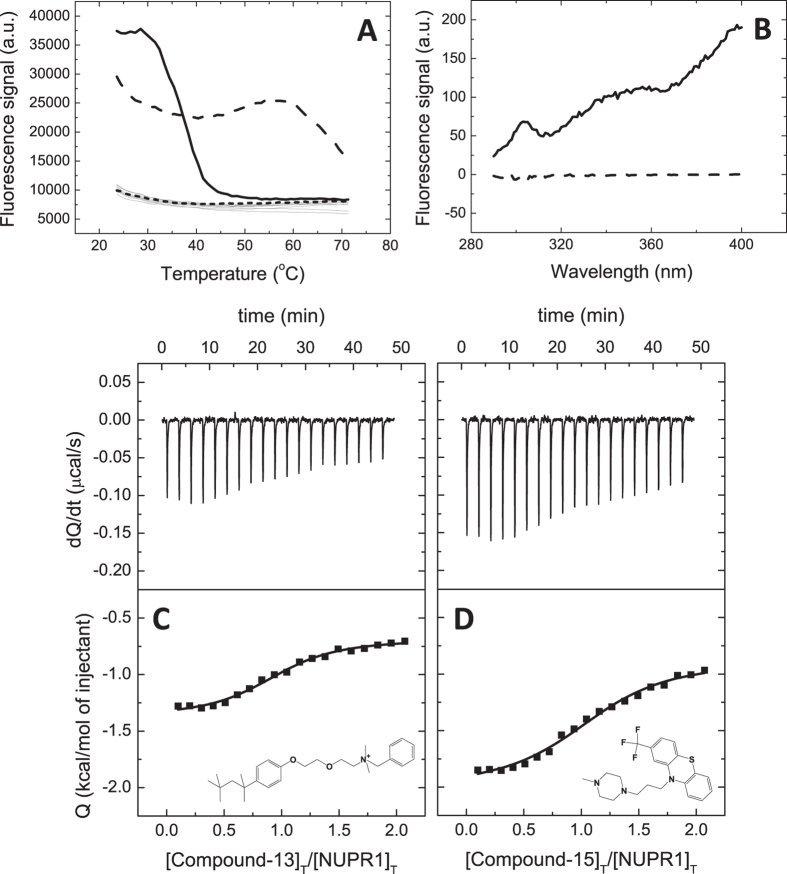
Screening and biophysical characterization of the binding of compounds to NUPR1. (**A**) Compounds interacting with NUPR1 were selected as those altering NUPR1 thermal denaturation profile; the most promising compounds (Compound-13 in dashed line and Compound-15 in continuous line), according to the subsequent assays, are shown. Typical denaturation profiles corresponding to control samples (NUPR1 with no compound) or compounds with no effect on NUPR1 are shown in dotted line or gray lines, respectively. (**B**) Difference spectra for Compound-13 (dashed line) and Compound-15 (continuous line) complexes. (**C**,**D**) Calorimetric titrations for Compound-13 (**C**) and Compound-15 (**D**) interacting with NUPR1. Thermograms (upper panels) and binding isotherms (lower panels) are shown. Non-linear fits according to a model considering a single ligand binding site (continuous lines) and molecular structures are shown.

A molecular screening *in vitro* based on thermal denaturation of NUPR1 in the presence of a variety of potential ligands was performed (Supplementary Table S1) using a collection of 1120 compounds (Prestwick Chemical Library). All compounds are FDA-approved drugs for a therapeutic indication, exhibiting high chemical and pharmacological diversity, as well as good bioavailability and safety in humans. Fifteen compounds, from now on named Compound-1 to Compound-15 (Table 1), were selected and identified as those inducing significantly different temperature denaturation profiles in NUPR1, compared to control sample (NUPR1 with no compound added) (Fig. 1A). The known therapeutic indication for each of the 15 compounds is reported in Supplementary Information (Table S2).

### Interaction between NUPR1 and selected compounds: Isothermal titration calorimetry

Ligand-induced stabilization of NUPR1 by the selected compounds represents an indirect piece of evidence for their interaction with NUPR1. Although ligand binding affinity and protein structural stabilization are intimately related, there is no direct correlation (that is, compounds exhibiting the same affinity do not necessarily induce the same stabilization effect). Thus, protein stability increments are not useful to rank ligand binding affinities; furthermore, the increased stability observed upon thermal denaturation may be the result of unspecific interactions between the ligand and the protein. Therefore, association constants of the selected compounds were directly determined using ITC. We were able to obtain calorimetric titrations for all of them (except for Compound-11, at the conditions tested) (Fig. 1C,D and Table 1). Dissociation constants were in the low micromolar range, indicating that these compounds would represent a good starting point for further affinity optimization.

### Interaction between NUPR1 and selected compounds: Fluorescence spectroscopy

As another piece of evidence for the direct interaction between NUPR1 and the Compounds, difference fluorescence spectra were determined for the NUPR1:Compound complexes. Difference spectra were obtained by subtracting the sum of the spectra for the individual components (NUPR1 or Compound) from that of the complex (NUPR1:Compound) at the same concentrations. A non-zero difference spectrum within the experimental error (that is, the spectrum of the complex is different to the sum of the individual spectra) reflected changes in the environment of aromatic residues in NUPR1, and, therefore, the interaction of compounds with NUPR1 (Fig. 1B).

### Defining the binding regions of the compounds in NUPR1

**Figure 2:**
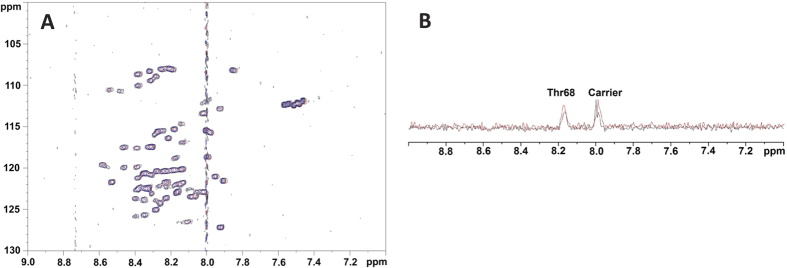
NMR screening of compounds to NUPR1. (**A**) 2D ^1^H-^15^N HSQC spectra of isolated NUPR1 (red) at 100 μM; NUPR1 and Compound-9 (black) (100:400 μM); and NUPR1 and Compound-15 (blue) (100:400 μM). (**B**) Rows from the ^1^H-^15^N HSQC spectra corresponding at the ^15^N chemical shift of Thr68 for isolated NUPR1 (red) and NUPR1 with Compound-15 added (black). The signal at 8.00 ppm appearing in both rows corresponds to the carrier position. Experiments were acquired at 25 °C and pH 4.5.

Next, we proceeded to identify the binding region(s) of NUPR1. Binding can be characterized by using either NMR chemical shift perturbation or variations in signal broadening of resonances of the 2D ^1^H-^15^N- heteronuclear single quantum coherence (HSQC) spectra. Addition of any compound to NUPR1 did not induce a change in the chemical shifts of any cross-peak. Since from the above experiments we already know that there is intermediate-to-slow between NUPR1 and the Compounds, this reveals that the exchange rate of complex formation is intermediate-to-slow within the NMR time scale, and broadening variation in the cross-peaks should be observed. Furthermore, these results indicate that the protein remained mainly disordered within the NMR time scale even after the binding occurs, and the effects observed in the thermal denaturation experiments must be local and restricted to particular polypeptide patches. Representative 2D HSQC data for two compounds (Compound 15 and Compound 9) are reported in Fig. 2A, showing the absence of changes in chemical shifts for any of the signals. It is important to note at this stage that changes in chemical shifts were observed in other studies describing interactions between small molecules and IDPs or intrinsically disordered regions of proteins^21^,^22^, but these changes were always very small. In particular, in one of the described examples the variations were only important in the ^1^H dimension^21^, and not in the ^15^N one. Moreover, it is important to note that in all these studies the amount of added ligand was always larger than that of the IDP, to ensure complex formation.

Close inspection of the rows, residue-by-residue, of the HSQC spectra for all compounds revealed non-uniform small variations in the broadening of the signals for some residues (Table 1). The broadening is caused by an exchange of compound molecules between the free and the NUPR1-bound state that is intermediate within the NMR time scale. The row corresponding to the ^15^N chemical shift of Thr68 of NUPR1 is shown in Fig. 2B in the absence or in the presence of Compound-15; clearly, it can be seen that for this residue there is a decrease in the intensity of the signal upon addition of the compound. The variations in all residues were very small, but fairly consistent among a restricted number of protein residues across several of the ligands; only Compound-1 did not show any difference in the broadening of any of the cross-peaks (Table 1). The fact that the variations, although being small, were observed for the same (or close in the primary structure) protein residues suggests that the binding mechanism is specific.

We believe that NUPR1 remained mainly disordered because of the absence of significant chemical shift changes in any resonance (Fig. 2A). We also attempted to acquire CD spectra of the complexes, but unfortunately the presence of dimethyl sulfoxide (DMSO, where the compounds were dissolved), which absorbs strongly at wavelengths below 225 nm, precluded any reliable measurement.

### Sequence-based analysis of the binding features of NUPR1

An *in silico* analysis of the binding properties of NUPR1 was performed following a two-part approach. First, a bioinformatic investigation of the protein sequence was carried out and, second, the structure of complexes with the selected compounds were modeled.

**Figure 3:**
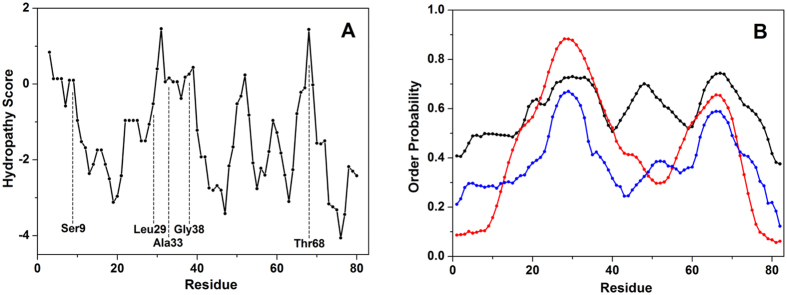
Properties of NUPR1 main chain as a function of residue number. (**A**) Hydrophobicity according to the scale of Kyte and Doolittle, calculated considering a window size of 5 residues. (**B**) Probability of conformational stability obtained by predicting the S^2^ order parameter of backbone N-H groups (black line) through DynaMine^24^,^25^ and by using PrDOS^26^ and DISOclust^27^,^28^ methods (red and blue line, respectively).

Figure 3A shows the hydropathy plot of NUPR1 as a function of residue number, calculated according to the hydrophilicity scale of Kyte-Doolittle^23^. A 5-residue window was used, which evaluates the local hydrophobicity around each amino acid by considering also the contribution of the two adjacent residues from each side along the sequence. The most prominent peaks correspond to residues whose resonances were affected by the binding of Compounds to NUPR1 (Table 1), with an offset of two residues at most. The two highest hydropathy scores correspond to residue 31, i.e. in between Leu29 and Ala33, and to Thr68. Other two peaks in the hydropathy pattern are found for residues 8–9 and 39, which account for Ser9, Ala10 and Gly38. These findings, together with our NMR studies, reveal that hydrophobicity is a main determinant for ligand association to NUPR1. However, it is important to stress out that the small variations in the NMR residues were not observed for all the amino acids involved in the theoretically identified hydrophobic patches, but only in a small, restricted subset of these. Thus, we concluded that the binding is occurring through the hydrophobic regions, but the results suggest that it is specific.

Predictions of the degree of order along the primary structure of NUPR1 were obtained with three different methods, all based solely on the knowledge of the protein sequence (Fig. 3B). Order probability values span from 0, representing a highly dynamic protein residue, to 1, indicating a complete local stability. DynaMine^24^,^25^ was used to predict the S^2^ order parameter (Fig. 3B, black line) for backbone N-H groups, which gives an estimate of likelihood of the protein chain flexibility. Although no residue is found in a stable arrangement (S^2^ ≥ 0.75), conformations for residues 26–37, 47–51 and 63–71 are classified as context-dependent (0.65 < S^2^ < 0.75). Additional calculations carried out by using PrDOS (Fig. 3B, red line), which combines local information on the protein sequence with a template-based prediction^26^, and DISOclust (Fig. 3B, blue line), which correlates protein disorder with per-residue errors in multiple fold recognition models^27^,^28^, consider the main chain region 47–51 as disordered (regions with probability < 0.5 are considered inherently disordered). Therefore, although using different strategies and not being reciprocally normalized, all predictors agree that only two regions, one including residues Leu29 and Ala33 (and marginally Gly38) and the other Thr68 (and marginally His62), are prone to become ordered under favorable conditions.

We also observed that the same regions of NUPR1 have a high probability of ligand binding through disorder-to-order transition (Supplementary Fig. S1) calculated by using other computational tools^29^,^30^,^31^, although not specifically designed to predict the association of small compounds.

Thus, to sum up, all the theoretical predictions based on the primary structure of the protein suggest that there are two main regions showing a high hydrophobicity, a certain grade of order, and a possible intrinsic tendency to be involved in binding to other molecules.

### Modeling the structures of NUPR1 complexes with the Compounds

As a second step in the *in silico* analysis, we tried to determine models of the structures of NUPR1 with the fifteen Compounds. As in the case of the previous theoretical predictions, the models were obtained blindly, i.e. without using any of the information provided by ITC and NMR.

**Figure 4:**
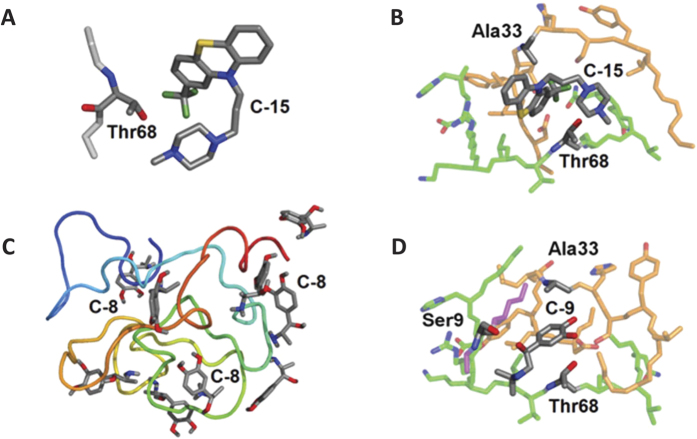
Docking of compounds to NUPR1. (**A**) Binding mode of Compound-15 with the side chain of Thr68–a portion of the protein main-chain (light gray) is also shown. (**B**) A transient protein pocket modelled by combining binding modes of Compound-15 with NUPR1 main chain portions including residues 27–40 (orange) and 62–72 (green). (**C**) Numerous binding modes of Compound-8 with the protein main chain (N and C terminus in blue and red, respectively). (**D**) A transient protein pocket of Compound-9 with NUPR1 main chain portions including residues 8–11 (purple), 27–40 (orange) and 60–72 (green). PyMol^57^ was used for all displays; hydrogens and protein main-chain oxygens are not shown, and protein backbone nitrogens are colored only for labelled residues.

A large set of different protein structures consistent with the hydrodynamic radius measured in the diffusion-ordered spectroscopy (DOSY) NMR experiments (22 ± 3 Å) were modelled by collapsing the extended protein chain in molecular dynamics (MD) simulations. Although it was not possible to prove convergence of sampling in our MD runs, neither that the selected protein conformations form a representative simulation ensemble, these structures provide some insight into the preferred conformation of NUPR1 in solution, and were used to determine potential binding locations for the compounds through molecular docking. The most favorable binding energies (−6.5 kcal/mol) were obtained for Compound-15 (see Fig. 4A) and Compound-2 interacting with the side chain of Thr68.

Other compounds (e.g., Compound-6, Compound-8 and Compound-9) showed a lower affinity (Fig. 4C). For some of them, the calculated binding energy was so low (−3.0 kcal/mol) that it could be reconciled with the experimental data only by assuming that multiple residues (including Ala33, Ser9, Ala10) cooperate in binding to the ligand. This was verified by modeling some *ad hoc* protein pockets (Fig. 4B–D), which showed an affinity increase of up to 3 kcal/mol. On the other hand, even in the most favorable case (i.e., Compound-15 and Compound-2), the binding energy could be reduced in the same amount if the ligand is docked at the same locations, but only a smaller portion (5–10 residues) of the protein backbone around the binding residues is included in the calculation. Overall, these results indicate that, although Thr68 is the preferred binding residue in NUPR1, not only a particular residue is important in providing the anchoring site for the corresponding compound, but also the concomitant presence of other nearby amino acids.

### The compounds hamper the interaction of NUPR1 to its natural partner MSL1 *in vitro*

Ligand binding to a given protein is a prerequisite for the ligand to modulate the biological activity of that protein. However, binding is not necessarily linked to having a modulatory effect and, consequently, phenotypic assays are needed in order to assess the potential bioactivity of compounds selected from biophysical or computational screenings. The above results have shown that several compounds can interact with NUPR1, but do the compounds exhibit any biological activity (e.g., alter any property of pancreatic cells)? And, more interestingly, is any of these compounds capable of interfering with an interaction between NUPR1 and a natural partner *in cellulo*?

**Figure 5:**
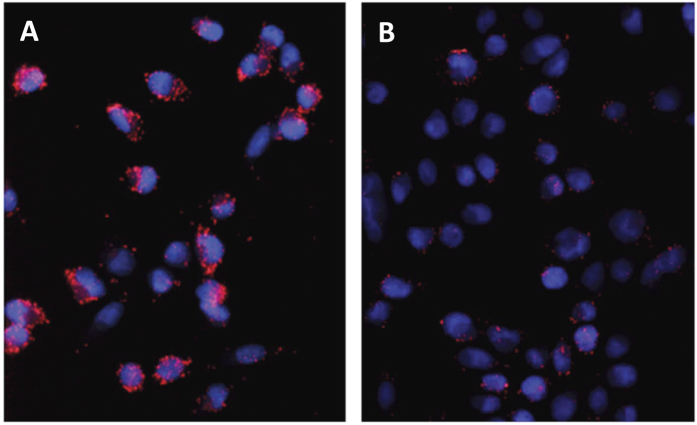
PLA of NUPR1 and MSL1. Cells were plated on coverslips and transfected with pcDNA3-NUPR1-Flag and pcDNA4-MSL1-V5 constructs. After one day, cells were treated simultaneously with Oxaliplatin (10 μM) to induce DNA damage (thus promoting MSL1-NUPR1 interaction), and (**A**) DMSO or (**B**) Compound-15 (in DMSO); PLA was performed 24 h later as described in the Methods section. The 40x magnification was analyzed with ImageJ to count the number of red dots which represent NUPR1/MSL1 binding. The reduction in the number of red fluorescent dots is proportional to the inhibition of MSL1-NUPR1 interaction by Compound-15.

To answer the first question, we carried out wound-healing (Supplementary Fig. S2) and clonogenicity assays (Supplementary Fig. S3). Our results show that the presence of any compound affected both features of human pancreatic cancer cells MiaPaCa-2. Although all compounds reduced colony formation in MiaPaCa-2 cells, Compound-13 and Compound-15 completely inhibited colony formation at 10 μM. To address the second question, since NUPR1 and MSL1 interact in the cell in response to induced DNA-damage^18^,^19^, we monitored such interaction both in the presence and in the absence of compounds, by using MiaPaCa-2 and proximity ligation assay (PLA) after Oxaliplatin-induced DNA-damage as experimental approach (in response to Oxaliplatin-induced DNA damage, MSL1 and NUPR1 interact to establish a DNA-repair complex^19^). In the PLA technique transfected MSL1 and NUPR1 proteins were tagged with antibodies against their respective tags (V5 and Flag), followed by ligation and amplification using fluorescent probes as previously described^19^. When binding occurs between them a considerable number of fluorescent dots within the transfected cells is observed. The treatment of MiaPaCa-2 cells with the Compounds counteracted NUPR1 and MSL1 interaction in a dose-dependent manner (in the range of 1 to 20 μM of final Compound concentration) (Fig. 5). The ligands bound to NUPR1 and hampered its interaction with MSL1 in cell-signaling, suggesting that MSL1 and the Compounds compete for the same binding-site region of NUPR1.

### Treatment of PDAC cells with Compound-15 mimics NUPR1-deficiency

**Figure 6:**
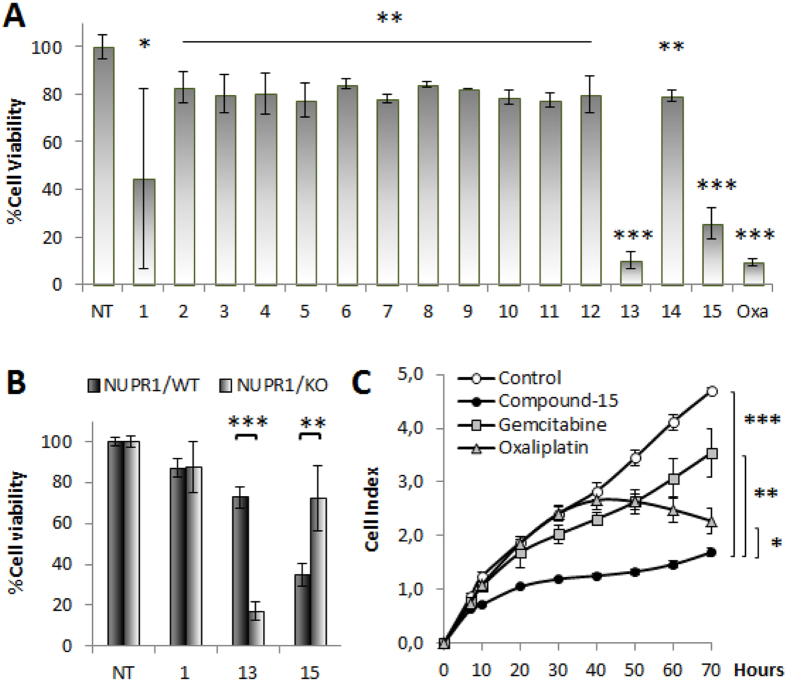
Compound-15 inhibits cell viability in a NUPR1-dependent manner. (**A**) MiaPaCa-2 and (**B**) primary murine cell lines genetically modified for not expressing NUPR1 (KO) regarding WT were seeded in 96-well plate (10000 cells/well) and treated with 10 μM of each Compound for 6 days. Error bars are standard deviations from 3 independent measurements. (p-value: * ≤ 0.1; ** ≤ 0.05; *** ≤ 0.0001) (**C**) iCELLigence system allowed us to follow MiaPaCa-2 cells proliferation in real-time and to observe the early efficiency of Compound-15 regarding effects of currently used chemotherapies (p-value * ≤ 0.1; ** ≤ 0.05; *** ≤ 0.001). The lines were drawn to guide the eye (NT means no treated and Oxa stands for Oxaliplatin). The error bars are standard deviations from 3 independent measurements.

NUPR1-deficiency in PDAC cells inhibits cell-growth and cell-migration, induces senescence, and decreases chemoresistance^13^. We hypothesized that a NUPR1 inhibitor must reproduce these effects in a NUPR1-dependent manner^13^. We performed a cell viability assay in 96-well plate and treated cells during 6 days with 10 μM of each compound (Fig. 6A). Compound-13 and Compound-15 showed higher stabilities than the rest of the compounds, and they were very efficient in diminishing cell viability (10 ± 3% and 26 ± 7%, respectively; p-value ≤ 0.01); in fact, these values are similar to those obtained with Oxaliplatin (10 ± 2%; p-value ≤ 0.01).

To confirm that this effect is NUPR1-dependent we performed a similar experiment using *NUPR1* knocked-out (KO) cells obtained from a mouse model of PDAC, which is genetically modified for not expressing NUPR1, and its wild-type (WT) counterpart. We observed an important difference between Compound-13 and Compound-15 suggesting a variation in the specificity of each molecule for NUPR1 (Fig. 6B). Whereas Compound-15 reduced cell viability to 35% (±6%; p-value ≤ 0.01) in WT, in KO cells we observed 72% (±16%; p-value ≤ 0.01) of viability. On the contrary, Compound-13 showed a high efficiency in KO cells regardless of NUPR1 expression (17 ± 4%; p-value ≤ 0.01). These differences strongly suggest that although Compound-15 shows a NUPR1-independent killing effect (around of 28%), a significant NUPR1-dependent effect is observed (around 37%). In contrast, Compound-13 shows a great NUPR1-independent effect and it was therefore discarded as a NUPR1 targeting drug candidate.

We also used the impedance iCELLigence system to monitor MiaPaCa-2 cells proliferation in real-time upon 6 days of treatment with Compound-15 (Fig. 6C). Compound-15 induced stronger growth-arrest, immediately after addition (p-value ≤ 0.01), and stronger than Oxaliplatin or Gemcitabine outcomes (p-value ≤ 0.05). These results indicate a more rapid effect compared to standard drugs, even if the final cell number was comparable.

Furthermore, we verified that the compounds decreased the spatial migration of MiaPaCa-2 cells. The most significant results were obtained with Compound-13 and Compound-15 (Supplementary Fig. S3). For the latter, cells migrated only 25% of the distance compared to Compound-free cells after 48 h, whereas Compound-13 treatment inhibited migration almost completely, inducing cell morphology modification.

Trying to get a deeper insight into the action of Compound-15 on NUPR1, we assessed whether the combination with standard chemotherapies could modify cell sensibility, and if so, we determined the IC_50_ value for each molecule (Supplementary Fig. S4). For both Gemcitabine and Oxaliplatin, IC_50_ in MiaPaCa-2 cells substantially decreased upon addition of 10 μM of Compound-15. In our hands, 60 nM of Gemcitabine reduced cell viability to one half (41 ± 0.4%), whereas a similar result was obtained with 4 nM of Gemcitabine in combination with 10 μM of Compound-15 (55 ± 6%; p-value ≤ 0.05). On the other hand, IC_50_ for Oxaliplatin was 4 μM (53 ± 0.4%; Supplementary Fig. S4), but together with 10 μM of Compound-15, just 15 nM was enough to reach the IC_50_ (57 ± 3%; p-value ≤ 0.01).

Finally, we tested the effect on senescence by measuring β-galactosidase activity. MiaPaCa-2 cells were treated with Compound-15, and its effect was compared with that of a specific siRNA targeting NUPR1 mRNA (Supplementary Fig. S5). A similar increase in blue cells in MiaPaCa-2 cells treated with Compound-15 (10 μM) was found, suggesting that Compound-15 inactivated NUPR1 leading to senescence.

In summary, Compound-15 interfered in the NUPR1-MSL1 interaction and inhibited cell growth, cell migration, induced cellular senescence, and decreased chemoresistance mimicking NUPR1-deficiency.

### NUPR1 inhibition with Compound-15 stops tumor progression

**Figure 7:**
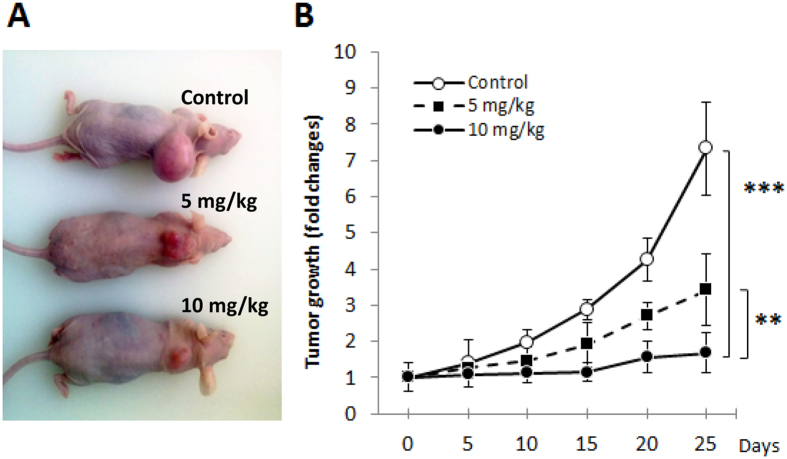
Compound-15 stops growth of human pancreatic tumors xenografts. (**A**) Representative images of tumor xenografts in mice treated with different concentrations of Compound-15 after 4 week-treatment. Size of tumors exponentially increased with time in control mice, whereas they are constant with Compound-15 treatment. (**B**) Fold change of tumor growth for each group of mice treated for 4 weeks with different concentrations of Compound-15 (control, 5 mg/kg, 10 mg/kg). Analysis of variance for repeated measurements indicated that the treatment was statistically significant p-value: ** ≤ 0.05; *** ≤ 0.01 compared to placebo.

Altogether, these results encouraged us to test Compound-15 *in vivo* with human PDAC-derived xenografts implanted into immunodeficient mice. PDAC-derived cells were inoculated sub-cutaneously in NMRI-Nude 8-week old mice. When tumors reached 400 mm^3^, we started a daily treatment for 4 weeks with two different concentrations, either low (5 mg/kg) or high (10 mg/kg), in two separate groups, and a third one (control) receiving an equivalent volume of vehicle. Tumor volumes increased in an exponential manner during two weeks (1530 ± 184 mm^3^) in control mice (Fig. 7). In contrast, with the lower dose of Compound-15, the tumor volume increased only 50% compared to the control during the same period (767 ± 196 mm^3^; p-value ≤ 0.01), and at higher Compound-15 dose the tumor growth was rapidly, and almost completely, stopped (558 ± 152 mm^3^; p-value ≤ 0.01), even after 4 weeks of treatment (Fig. 7). In conclusion, a daily treatment with Compound-15, at a concentration of 10 mg/kg, was able to stop growth of the PDAC tumor xenografted in immunodeficient mice.

## Discussion

### A proof-of-concept approach for targeting drugs against IDPs remaining disordered upon binding to their natural partners

We have developed an approach to characterize and tackle the druggability of an IDP that relies on the combination of biophysical techniques, *in silico* calculations and *in vivo* studies in cells and organisms. The NUPR1 remains disordered upon binding to MSL1^19^ and DNA^9^, forming “fuzzy” complexes with those biomolecules. We hypothesized that the ability of NUPR1 to form disordered, or fuzzy, complexes with other biomolecules would confer the ability to bind small molecules through complexes with a similar degree of disorder.

The most immediate outcome of our work is the identification of a compound, Trifluoperazine, active against PDAC. In addition to its efficacy and specificity, this newly discovered compound can also be used in combination with standard anti-cancer drugs (Oxaliplatin and Gemcitabine). More generally, we have proved it is possible to identify a low-molecular-weight compound against an IDP inhibiting its interactions with other proteins (that is, a twofold challenge: blocking a PPI between IDPs). Thus, we have shown that disordered interfaces between IDPs are “druggable-targets”, taking NUPR1, an IDP involved in several signaling pathways, as a proof-of-concept.

Designing low-molecular-weight drugs for inhibiting PPIs in IDPs implies several challenges: (1) absence of well-defined protein structures for molecular modelling and structured-based development; (2) large PPI interfaces to be obstructed by small protein-drug interaction interfaces; and, (3) multiplicity of PPIs to be inhibited, since IDPs are usually involved in numerous biomolecular interactions (“moonlighting”). Three approaches in drug-development towards IDPs have been considered so far. The first one exploits the fact that PPIs involving IDPs can be modulated by organic compounds because an IDP undergoes, very often, a disorder-to-order transition upon binding to its (usually ordered) partner^21^,^32^,^33^; therefore, the organic compound is designed against the interface of the fully structured complex, and it competes for the same interacting surface as the natural protein partner. The second approach focuses on stabilizing the unfolded conformation of the IDP, and thus the compound binds and shifts the conformational equilibrium towards the unfolded species^34^,^35^,^36^. In the third approach, a small molecule is selected to bind the specific regulatory disordered region of an otherwise well-folded protein, and inhibits the enzyme through an allosteric mechanism^22^. Regarding the protein used in this work, the molecular partner considered for NUPR1 is MSL1, which is also an IDP and, interestingly enough, both of them interact keeping their disordered conformations^19^. Thus, we have developed a fourth approach for designing drugs against IDPs (which includes some features of the previous methods): a small molecule forms a “fuzzy complex” with the target IDP and competes for the same polypeptide region, as the natural biomolecular partner of the IDP does.

The final biological impact of Compound-15 (i.e., tumor suppression in PDAC-xenografted mice) suggests that our global strategy for identifying NUPR1 binding compounds is correct. However, although we have rationally selected Trifluoperazine as an interacting ligand for NUPR1 by using biophysical and computational tools, we cannot exclude that its biological activity here reported might rely on binding to other different targets (as suggested by the results reported in Fig. 6), due to the ability of this compound to affect various tumoral pathways^37^. We believe that due to the inherent difficulty of proving the binding of a small molecule with a low-populated protein conformation (either *in vitro* or in the cell), appropriately designed cellular or *in vivo* phenotypic assays for assessing biological activity are a key step for detecting the interaction between the compound and its protein target.

### The molecular basis of the binding regions of NUPR1

It has been previously shown that the binding region of NUPR1 towards MSL1 comprises, among others, Leu24-Asp28, Tyr30, Ala33-His34, and Lys65-Thr68 polypeptide patches^19^. All these residues are far away in the primary structure of the protein, but they must be close together upon binding to MSL1, although we do not know the kind of secondary and/or tertiary structure they are engaged on. It is interesting to note that some of Asp and Glu residues in those regions are close enough in conformations populated by NUPR1 at high NaCl concentrations^20^.

**Figure 8:**
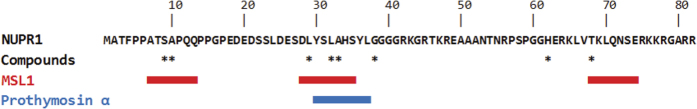
The “hot-spot” regions of NUPR1. The sequence of human NUPR1 is shown at the top. Residues whose broadening of cross-peaks of their amide resonances were affected by the presence of any of the fifteen compounds are indicated by an asterisk. The region affected by binding of prothymosin α was monitored by fluorescence changes^14^. The regions affected by binding to MSL1 were monitored by NMR chemical shift changes^19^.

All the selected organic compounds studied in this work induced slight broadening of NMR cross-peaks of residues in the above regions (Table 1 and Fig. 2B), indicating that they interacted at the same patches as the natural binding partner, without inducing any rigid structural order in NUPR1. It is important to remark here that the natural partner, MSL1, does induce small chemical shift changes in the resonances of those residues of NUPR1 (see Supplementary Information in ref. 19). In addition, in our MD simulations the nearest regions to the key residue Thr68 are Leu37 and Gln31, which are adjacent to Tyr36 and Tyr30 (the sole fluorescent residues in NUPR1); thus, the simulation data explain the changes in the experimentally observed fluorescence spectra of NUPR1 upon addition of many of the Compounds (Fig. 1). Both tyrosines are also involved in the binding to prothymosin α^14^, and previous MD simulations suggest they also intervene (together with nearby regions) in the binding to DNA^38^. Thus, all the different ligand species (either single large biomolecule or small organic compounds) seem to bind to the same regions of NUPR1, as shown in Fig. 8.

In addition, our experimental and theoretical analyses reveal that hydrophobicity is a main determinant for the Compound association to NUPR1, and that the protein residues involved have a partially restricted conformation in solution, although we do not know the kind of secondary structure they are involved in. All these results suggest that the mechanism of action of the Compounds against NUPR1 can be explained in terms of a competition for the same hydrophobic, locally-restricted, hot-spot region. An additional hint about the importance of hydrophobic interactions in the binding of Compound-15 comes from the thermodynamic binding profile of the selected compounds; in particular, Compound-15 exhibits an entropically-driven binding with a small enthalpic contribution (Δ*H* = −1.1 kcal/mol) and a large favorable entropic contribution (−*T*Δ*S* = −6.1 kcal/mol) to the Gibbs energy of binding (Δ*G* = −7.2 kcal/mol) (Fig. 1). A very similar binding profile is found for Compound-13 (Δ*G* = −7.4 kcal/mol, Δ*H* = −0.7 kcal/mol, −*T*Δ*S* = −6.7 kcal/mol) (Fig. 1) and the rest of the selected compounds. The large favorable entropic contribution reflects a considerable entropy gain from desolvation of hydrophobic surfaces upon binding and a small entropy loss stemming from the small ordering associated with the formation of the disordered or “fuzzy” NUPR1-compound complex. The design of small drugs against other IDPs also suggests that hydrophobic interactions are mainly involved in the binding and, as it happens in NUPR1, aromatic residues seem to be critical in p27^21^. In our docking simulations, in addition to hydrophobicity, interaction with the Compounds is also mediated by electrostatic contributions and, in a few of the Compounds, by the presence of hydrogen-bond donors and acceptors, as it has been also observed in the binding of small molecules to the disordered region of Myc^35^.

MSL1 and NUPR1 interact with an association constant in the range of 3 μΜ^19^ and they interact with DNA^19^ with an affinity similar to those of the selected compounds for NUPR1 (Table 1). Thus, based on our quantitative measurements, the Compounds are capable to compete for the same NUPR1-binding site, with an affinity similar to that of MSL1. From all our experimental evidence about the binding of NUPR1 to its natural partners^9^,^14^,^19^ (and this work), we believe that NUPR1 remains disordered or “fuzzy” upon binding to any molecule (organic or biomolecule), and such disordered regions facilitate binding, in contrast to recent experimental findings in which “fuzzy” regions seem to hamper the binding^39^. We also hypothesize that larger molecules with a high hydrophobicity (and thus more capable of being involved in the interactions with the NUPR1 regions nearby Thr68 and around residues 28–38) will be better templates for binding and blocking NUPR1 function.

Finally, we suggest that the binding site of NUPR1 is formed by malleable, highly mobile regions that can accommodate the natural partners and organic molecules with, at least, the same degree of hydrophobicity. Thus, our model protein does not only describe a new approach to drug-selection against IDPs, but also pinpoints that the mode of action of the drugs against IDPs can be very different depending of the targeted protein, as it happens with well-folded proteins^3^.

## Methods

### Materials

Deuterium oxide and IPTG were obtained from Apollo Scientific (Cheshire, UK). Sodium trimethylsilyl [2,2,3,3-^2^H_4_] propionate (TSP), ANS, deuterated acetic acid and its sodium salt were obtained from Sigma Aldrich (Madrid, Spain). Dialysis tubing, with a molecular weight cut-off of 3500 Da, was from Spectra/Por (Spectrum Labs, Shiga, Japan). Standard suppliers were used for all other chemicals. Water was deionized and purified on a Millipore system.

The compounds of the chemical library (Prestwick Company, Illkirch, France) were supplied dissolved in DMSO 100% at a concentration of 4 mM. According to the manufacturer, the compounds are FDA-approved drugs selected for their high chemical and pharmacological diversity. In addition, information on their bioavailability, as well as toxicity and safety, in humans is available.

The *NUPR1* vector and construction have been described elsewhere^9^.

### Protein expression and purification

NUPR1 was produced and purified from transformed *E. coli* grown in LB media as previously described^9^. For the production of ^15^N- labelled samples, the cells were grown in M9 minimal media, supplemented with vitamins, and purified as the protein obtained from *E. coli* grown in LB media^9^.

### Experimental screening

Ligands for NUPR1 have been identified by an experimental screening procedure based on a thermal-shift assay (ligand-induced stabilization against thermal denaturation) similar to that employed previously for identifying small-molecule compounds acting as inhibitors of NS3 protease from hepatitis C virus^36^, inhibitors of *Helicobacter pylori* flavodoxin^40^ and pharmacological chaperones of human phenylalanine hydroxylase^41^.

Thermal denaturations of NUPR1 were performed in a FluoDia T70 Fluorescence Microplate Reader (Photon Technology International, North Edison, NJ). Solutions containing 4 μM NUPR1, 100 μM compound (2.5% residual final concentration of DMSO) and 100 μM ANS in 20 mM sodium phosphate pH 7 with a 100 μL total volume were dispensed into 96-well microplates (ThermoFast 96 skirted plates, from Thermo Scientific, Madrid, Spain). Solutions were overlaid with 20 μL of mineral oil to prevent evaporation and incubated at 25 °C for 30 minutes before measurement.

Unfolding curves were registered from 25 to 75 °C in 1 °C steps following ANS emission fluorescence (excitation and emission wavelengths of 395 and 500 nm, respectively), which greatly increases when this probe binds to hydrophobic regions in the protein exposed to the solvent upon thermal unfolding. The system was allowed to equilibrate at each temperature for 1 min before each fluorescence acquisition (an operational heating rate of 0.25 °C/min approximately). Control experiments with NUPR1 samples with/without DMSO were routinely performed in each microplate.

Hits were identified as those compounds shifting the temperature for maximal slope towards higher temperatures, compared to the internal controls in each microplate, thus inducing a stabilizing effect on NUPR1 and potentially capable of inhibiting any subsequent protein-NUPR1 interactions. Furthermore, compounds inducing a markedly different denaturation pattern compared to free NUPR1 were also selected.

### Isothermal titration calorimetry (ITC) assays

Ligand binding to NUPR1 was determined with a high sensitivity isothermal titration calorimeter Auto-iTC200 (MicroCal–Malvern Instruments, Malvern UK). Protein samples and reference solutions were properly degassed and carefully loaded into the calorimetric cells to avoid bubble formation during stirring. Experiments were performed with freshly prepared buffer-exchanged protein solutions, at 25 °C in 20 mM sodium phosphate pH 7. NUPR1 20 μM solution in the calorimetric cell was titrated with 300–400 μM compound solution. A sequence of 19 injections of 2 μL volume was programmed with a stirring speed of 1000 rpm. The heat evolved after each ligand injection was obtained from the integral of the calorimetric signal. The heat due to the binding reaction was obtained as the difference between the reaction heat and the corresponding heat of dilution, the latter estimated as a constant value throughout the experiment, and included as an adjustable parameter in the analysis. Control experiments (compound injected into buffer) were performed under the same experimental conditions. The association constant (*K*_a_) and the enthalpy change (Δ*H*) of the binding reaction were obtained through non-linear regression of experimental data to a model for a protein with a single ligand binding site. Experiments were performed in replicates and data were analyzed using in-house developed software implemented in Origin 7 (OriginLab, Northampton, MA).

### Fluorescence measurements

Fluorescence spectra were collected in a Cary Eclipse spectrofluorometer (Varian–Agilent Technologies, Santa Clara, CA) interfaced with a Peltier-thermostated multicell holder. The slit widths were 5 nm for both excitation and emission wavelengths. Excitation wavelength was 280 nm. Emission spectra from 300 to 400 nm were acquired at 25 °C, in a 1-cm-pathlength quartz cell (Hellma Analytics, Müllheim, Germany). The NUPR1 concentration was 3 μM in buffer in 20 mM sodium phosphate pH 7, and 100 μM of Compound was added in each experiment. Difference spectra were obtained by subtracting the sum of the spectra for the individual components (NUPR1 and Compound) from that of the complex (NUPR1:Compound) at the same concentrations.

### NMR experiments

The NMR data were acquired at 25 °C, pH 4.5 (acetate buffer), on a Bruker Avance DRX-500 spectrometer equipped with a triple-resonance probe and z-gradients. All spectra were referenced to external TSP.

The 2D ^1^H-^15^N heteronuclear single-quantum coherence (HSQC) spectra^42^ were acquired in the phase sensitive mode for isolated ^15^N-labelled NUPR1 (100 μM) or ^15^N-labelled NUPR1 (100 μM) in the presence of the corresponding compound (400 μM). Similar protein/drug ratios have been used in other studies aimed to test druggability of IDPs^21^,^22^,^34^,^35^. Frequency discrimination in the indirect dimensions was achieved by using the echo/antiecho-TPPI method. The spectra were acquired with 1 K complex points in the ^1^H dimension, 128 complex points in the ^15^N dimension, and 200 scans. The carrier of the ^1^H dimension was set at 8.00 ppm, and that of ^15^N at 120 ppm. The spectral widths used were 10 and 35 ppm in the ^1^H and ^15^N dimensions, respectively. Water signal was suppressed with the WATERGATE sequence^43^. Data were zero-filled to double the number of original points in both dimensions, apodized with shifted squared sine-bell functions in the two dimensions and Fourier transformed with the program TopSpin 1.2. Assignments were taken from those previously reported^19^. The intensity of the signals from each row in the HSQC spectra was measured by using TopSpin 2.1 taking into account, as an internal reference, the intensity of the last residue of NUPR1. Only differences in intensity of a particular residue between spectra of the complex (NUPR1:Compound) and that of isolated NUPR1 larger than 10%, were considered significant.

The diffusion-ordered spectroscopy (DOSY) measurements were carried out at 25 °C, as described^44^. The methyl groups with chemical shifts between 1.0 and 0.70 ppm of NUPR1 were used for integration; the diffusion translational coefficient value is the average of two measurements. The gradient strength was calibrated by using the value of the diffusion translational coefficient for the residual proton water line in a sample containing 100% D_2_O in a 5-mm tube^44^. A 1% final concentration dioxane was added; the hydrodynamic radius, *R*_h_, of NUPR1 was obtained by assuming that the *R* of dioxane is 2.12 Å^45^. The value was 22 ± 3 Å, and it was used to compare with the gyration radii of the modeled molecules during the MD simulations.

### Computational methods

Protein and ligand structures were built by using VMD^46^ and UCSF Chimera^47^, respectively. NUPR1 was initially modelled as an extended chain, then collapsed in very short (15–30 ps) MD simulations *in vacuo*, and equilibrated for 80–200 ns in the presence of water (TIP3P model^48^) and five Cl^−^ counterions. A rhombic dodecahedron box with minimum distance 10 Å from the solvent was employed and periodic boundary conditions were applied. A set of 16 simulations were performed by using the GROMACS 4.5 package^49^ with AMBER ff99SB-ILDN force field^50^. Long-range electrostatic interactions were calculated through the particle-mesh Ewald method^51^,^52^. Sampling was performed in the isothermal-isobaric ensemble by using a velocity rescaling thermostat^53^ and Berendsen barostat^54^, with reference values of 300 K and 10^5^ Pa, respectively.

Protein structures were extracted from the simulation trajectory by using a cluster analysis, and used to perform docking experiments of compounds with AutoDock Vina^55^. A large variety of structures were obtained (8–32 structures, depending on the clustering procedure), allowing us an extensive investigation of the binding properties of NUPR1 in molecular docking. Full flexibility was allowed for each ligand in the docking calculations. In some cases of special interest, the possibility was tested of simultaneous interaction of a compound with multiple selected protein residues, each individually assessed as a binding location both in NMR and docking experiments, but distant one another in the NUPR1 structures obtained in simulation. Such occurrences were modelled by superimposing on the ligand the corresponding (end-capped) portions of the protein main chain. These complexes, which simulate the presence of a compound in a (transient) protein pocket, were equilibrated in short MD runs (2 ns) under the same conditions described above, by additionally using for the ligand the GAFF force field^56^.

### Animals

NMRI-Foxn1^nu^/Foxn1^nu^ mice were provided by Janvier Laboratories (Le Genest-Saint-Isle, France). Mice were kept within the Experimental Animal House of the Centre de Cancérologie de Marseille (CRCM) pôle Luminy. All experimental protocols were carried out in accordance with nationally approved guidelines for the treatment of laboratory animals. All experimental procedures on animals were approved by the Comité d’éthique de Marseille numéro 14. Ten millions MiaPaCa-2 cells were inoculated subcutaneously and mice were separated into 3 groups of 6 subjects each. Mice were treated daily with either physiologic serum, 5 mg/kg or 10 mg/kg of the compound when the tumor volume reached approximately 400 mm^3^. Every 5 days, the weight and the tumors volumes were measured. Mice were sacrificed after 35 days of treatment.

### Cell cultures

MiaPaCa-2 cells were obtained from ATCC (Manassas, VA) and maintained in Dulbecco’s Modified Eagle’s Medium (DMEM) (Invitrogen, Carlsbad, CA) supplemented with 10% FBS (Foetal Bovine Serum) at 37 °C with 5% CO_2_. MiaPaCa-2 cells were authenticated using Short Tandem Repeat analysis (LGC Standards GmbH, Wesel, Germany). INTERFERin reagent (Polyplus-transfection, Illkirch, France) was used to perform siRNA transfections according to the manufacturer’s protocol. Scrambled siRNA targeting no known gene sequence was used as negative control. The sequence of NUPR1-specific siRNA-*nupr1* r(GGAGGACCCAGGACAGGAU)dTdT was previously reported^10^. Knock-out cells were established in the laboratory from a mice model of pancreatic cancer, known as Pdx1-Cre-Kras^G12D^/INK4^flox/flox^ and for those *nupr1* was deleted. These cells have been supplied with DMEM (4.5 g/L) and Glutamax (Life Technologies, Carlsbad, CA), supplemented with 10% of FBS.

### Cell viability assays

Cells (10000 cells/well) were plated in 96-well plates with DMEM. Twenty-four hours later, the media were supplemented with 10 μM of Compound and were incubated for another additional six days. Each experiment was performed in duplicate and repeated at least three times. Cell viability was estimated after addition of the CellTiter-Blue viability reagent (Promega, Madrid, Spain) for 1 h according to the protocol provided by the supplier. Cell viability was measured on day 6 and normalized regarding untreated cells rates. Statistical significance was assumed at a p-value lower than 0.05.

### Real-time cellular iCELLigence assays

Cell proliferation was monitored in real-time using the iCELLigence system E-Plate (ACEA Biosciences, San Diego, CA). Forty thousand MiaPaCa-2 cells were seeded in each well with fresh DMEM. Seven hours later, medium was changed with 10 μM Compound-15, 60 nM Gemcitabine, 4 μM Oxaliplatin or combinatory treatments. Cells were incubated for another 3 days. The impedance value of each well was automatically monitored by the iCELLigence system for 3 days and expressed as a CI (cell index) value. Statistical significance was assumed at a p-value lower than 0.05.

### SA-β-galactosidase activity assays

Cells cultured on glass coverslips were tested for SA-β-galactosidase activity using the Senescence β-galactosidase Staining Kit (Cell Signaling, Danvers, MA) according to the manufacturer’s protocol. After 48 hours of treatment, cells were washed with phosphate buffered saline (PBS) 1x (Life Technologies, Carlsbad, CA), then fixed and stained with the provided reagents. The blue color is the reporter of senescence.

### Wound healing assays

Cells (10000 cells/well) were seeded in 24-well plates and grown to confluence. The monolayer of MiaPaCa-2 cells was then scraped with a sterile 200 μL pipette tip and washed three times with PBS to remove the detached cells before compound treatment. Either fresh medium with 10 μM of the corresponding compound were added or DMSO (as control). Cell migration to the wounded region was observed using an automated fluorescence inverted microscope Leica DMI 6000B with MetaMorph MM AF analysis software (Leica Microsystems, Wetzlar, Germany). Images were captured at 0, 24 and 48 h to monitor the wound healing process. The wound areas were measured using ImageJ software (NIH, Bethesda, MD). Comparisons of treatment outcome were tested for statistical significance by using the t-test. Statistical significance was assumed at a p-value lower than 0.05.

### Colony formation in soft agar assays

Colony formation assay was performed in order to measure the *in vitro* survival ability of a single cell to grow into a colony in an anchorage-independent growth environment. Briefly, after treating MiaPaCa-2 cells with either the compounds or DMSO (as control) for 24 and 48 h, they were seeded in DMEM complete media at a density of 7 × 10^3^ cells in 6-well plates containing a top layer of 0.7% agar and a bottom layer of 1% agar. Plates were incubated at 37 °C for 3 to 4 weeks and then stained with 0.2% crystal violet. Colonies were observed with a Leica DMI 6000B microscope and counted (those with more than 20 cells) using the MetaMorph MM AF analysis software. Comparisons of treatment outcome were tested for statistical significance by using the t-test. Statistical significance was assumed at a p-value lower than 0.05.

### Protein ligation (PLA) assays

MiaPaCa-2 cells were seeded on coverslips and transfected with 300 ng of DNA (NUPR1-Flag and MSL1-V5) using Fugene HDTM transfection reagent (Roche, Meylan, France). After one day, cells were treated simultaneously with Oxaliplatin (10 μM) to induce DNA damage (thus promoting MSL1-NUPR1 interaction as a DNA-repair complex^19^), and either DMSO or Compound. Cells were washed twice in PBS, fixed, permeabilized and saturated for 45 min before immunostaining with DuoLink kit (Olink Bioscience, Uppsala, Sweden) following the manufacturer’s protocol. Image acquisition was carried out on a Nikon Eclipse 90i fluorescence microscope (Nikon Instruments, Amsterdam, Netherlands). Images at 40x magnification were analyzed with ImageJ to count the number of red dots which represent NUPR1/MSL1 binding. The reduction in the number of red fluorescent dots is proportional to the inhibition of MSL1-NUPR1 interaction by Compound-15.

## Additional Information

**How to cite this article**: Neira, J. L. *et al*. Identification of a Drug Targeting an Intrinsically Disordered Protein Involved in Pancreatic Adenocarcinoma. *Sci. Rep.* **7**, 39732; doi: 10.1038/srep39732 (2017).

**Publisher's note:** Springer Nature remains neutral with regard to jurisdictional claims in published maps and institutional affiliations.

## Supplementary Material

Supplementary Information

## Figures and Tables

**Table 1:** Dissociation constants of the NUPR1-Compound complexes, and residues of NUPR1 with NMR-cross-peak broadening affected by binding.

	Compound	*K*_d_ (μM)^a^	Residues
1	Terfenadine	5.0	—
2	Fluphenazine dihydrochloride	2.0	Thr68
3	Caffeic acid	2.0	Ala33; Thr68
4	Reserpine	3.2	Thr68
5	(-)-Isoproterenol hydrochloride	3.9	Thr68
6	Flunarizine dihydrochloride	3.1	Ala33; Thr68
7	Halofantrine hydrochloride^b^	3.3	Thr68
8	Levonordefrin	1.5	Ala33; Thr68
9	(+)-Isoproterenol (+)-bitartrate salt	4.0	Ser9; Ala10; Leu29; Ala33; Gly38; Thr68
10	Pheniramine maleate	4.3	Ser9; His62; Thr68
11	Terconazole	—c	Thr68
12	Dihydroergotoxine mesylate	4.0	Leu29; Leu32; Gly38; Thr68
13	Benzethonium chloride	3.6	Thr68
14	Chlortetracycline hydrochloride	1.5	Ala33; Thr68
15	Trifluoperazine dihydrochloride	5.2	Ala33; Thr68

^a^Relative error is 20%.

^b^At the NMR concentrations, the compound precipitated.

^c^At the conditions tested, it could not be determined.
