# Supplementary material for: Identification of a Drug Targeting an Intrinsically Disordered Protein Involved in Pancreatic Adenocarcinoma
Source: Sci Rep. 2017 Jan 5;7:39732. doi: 10.1038/srep39732 (PMC5213423; doi:10.1038/srep39732)

SUPPLEMENTARY INFORMATION

Identification of a Drug Targeting an Intrinsically Disordered Protein Involved in Pancreatic Adenocarcinoma

Jose L. Neira, Jennifer Bintz, Maria Arruebo, Bruno Rizzuti, Thomas Bonacci, Sonia Vega, Angel Lanas, Adrian Velazquez-Campoy, Juan L. Iovanna and Olga Abian

SUPPLEMENTARY MATERIAL

Table S1. Small molecule screening data

**Category**	**Parameter**	**Description**
Assay	Type of assay	*In vitro*
	Target	NUPR1 (p8)
	Primary measurement	Detection of ligand-induced stabilization effect upon thermal denaturation (increase in T_m or altered thermal unfolding profile)
	Key reagents	ANS, 8-anilino 1-naphthalene sulfonic acid
	Assay protocol	See main text in Methods section
	Additional comments	
Library	Library size	1120 compounds
	Library composition	Compound at 4 mM in 100% DMSO
	Source	Prestwick Chemical Library (Prestwick Chemical, Illkirch, France)
	Additional comments	
Screen	Format	96-well plate
	Concentration(s) tested	100 µM at 2.5% DMSO
	Plate controls	NUPR1 with 2.5% DMSO and no compound
	Reagent/ compound dispensing system	Multichannel pipette

	Detection instrument and software	FluoDia T70 (Photon Technology International)
	Assay validation/QC	Control standard deviation and uniformity
	Correction factors	N/A
	Normalization	N/A
	Additional comments	
Post-HTS analysis	Hit criteria	$T_m > T_{m}$,control + 2°C; altered thermal unfolding profile
	Hit rate	1.25% (15 out of 1200)
	Additional assay(s)	Retest of initial hits, *in-vitro* and cell-based secondary assays (see Supporting Information)
	Confirmation of hit purity and structure	Compounds repurchased from Prestwick Chemical
	Additional comments	

Table S2. Therapeutic information for the 15 selected compounds from Prestwick Chemical Library.

	Compound	Therapeutic information
1	Terfenadine	A selective histamine H1-receptor antagonist devoid of central nervous system depressant activity. The drug was used for allergy but withdrawn due to causing long QT syndrome.
2	Fluphenazine dihydrochloride	A phenothiazine used in the treatment of psychoses. Its properties and uses are generally similar to those of chlorpromazine (antipsychotic medication, primarily used to treat psychotic disorders such as schizophrenia; other uses include the treatment of bipolar disorder, attention deficit hyperactivity disorder, nausea and vomiting, and anxiety before surgery)
3	Caffeic acid	A hydroxycinnamic acid with an antioxidant activity in vitro and in vivo. It shows immunomodulatory and anti-inflammatory activity.
4	Reserpine	An indole alkaloid with antipsychotic and antihypertensive activity. It has been used for the control of high blood pressure and for the relief of psychotic symptoms.
5	(-)-Isoproterenol hydrochloride	A synthetic sympathomimetic amine that is structurally related to epinephrine, but acts almost exclusively on beta receptors.
6	Flunarizine dihydrochloride	A selective calcium entry blocker with calmodulin binding properties and histamine H1 blocking activity. It is effective in the prophylaxis of migraine, occlusive peripheral vascular disease, vertigo of central and peripheral origin, and as an adjuvant the therapy of epilepsy.
7	Halofantrine hydrochloride	A drug used to treat malaria, related to the antimalarial drugs quinine and lumefantrine.

8	Levonordefrin	A norepinephrine derivative used as a vasoconstrictor agent.
9	(+)-Isoproterenol (+)-bitartrate salt	See Compound 5
10	Pheniramine maleate	A histamine H1 antagonist with little sedative action. It is used in treatment of hay fever, rhinitis, allergic dermatoses, and pruritus.
11	Terconazole	An anti-fungal medication primarily used to treat vaginal fungal infections.
12	Dihydroergotoxine mesylate	A mixture of the mesylates (methane sulfonates) of dihydroergocornine; dihydroergocristine; and the alpha- and beta-isomers of dihydroergocryptine. The substance produces a generalized peripheral vasodilation and a fall in arterial pressure and has been used to treat symptoms of mild to moderate impairment of mental function in the elderly.
13	Benzethonium chloride	Bactericidal cationic quaternary ammonium surfactant used as a topical anti-infective agent. It is an ingredient in medicaments, deodorants, mouthwashes, etc., and is used to disinfect apparatus, etc., in the food processing and pharmaceutical industry surgery, and also as a preservative. The compound is toxic orally as a result of neuromuscular blockade.
14	Chlortetracycline hydrochloride	A tetracycline antibiotic, the first tetracycline to be identified.
15	Trifluoperazine dihydrochloride	A phenothiazine with actions similar to chlorpromazine (see Compound 2). It is used as an antipsychotic and an antiemetic.

SUPPLEMENTARY FIGURE LEGENDS

Figure S1. Binding probability through disorder-to-order transition of NUPR1.

Probability of binding along the protein sequence obtained by using the computational predictor MoRFpred[29] (black line), which identifies short binding regions characterized by a combination of high hydrophobicity and order propensity, and ANCHOR[30,31] (red line), which uses energy estimations to detect main chain segments that can be stabilized in the presence of a molecular partner.

Figure S2. Wound healing assay of MiaPaCa-2 cells in presence of compounds.

(A) Migration of cells in the presence of each Compound expressed as percentage reduction in wound size (average distance between monolayers) as a result of cell migration after 24 h (black) and 48 h (grey). Bars indicate the ratio between the wound size at each time and the initial size. Error bars represent the SD (standard deviation) for n=3 experiments carried out in triplicate. Larger bars indicate reduced wound healing (reduced cell migration). $*p<0.05$ (the p-values were obtained using the Student's two-tailed, paired t-test with the control at the same time either 24 or 48 h). (B) Representative images of the process taken at initial time, at 24 and 48 h later, after treatment with 10 µM of the corresponding Compound.

Figure S3. Evaluation of the clonogenicity of MiaPaCa-2 cells in presence of compounds.

(A) Number of colonies in the presence of each compound at 10 µM (black) and 50 µM (grey). Error bars represent the standard deviation for n=3 experiments carried out in triplicate. (B) Representative images of the process.

Figure S4. Compound-15 decreases chemo-resistance in pancreatic cancer cell lines.

Chemograms have been made from MiaPaCa-2 cells seeded in 96-well plate (10000 cells/well) and treated with increasing amounts of Compound-15 (A), Gemcitabine (B) or Oxaliplatin (c) from 1 nM to 1 mM with or without adding 10 µM of Compound-15 for 3 days. The lines were drawn to guide the eye. Error bars represent the standard deviation from 3 independent experiments.

Figure S5. Compound-15 counteracts cell adaptive responses usually triggered by NUPR1.

(A) Microscopic view (Gx20) of MiaPaCa-2 cells stained with SA-βGal (blue colour) representative of senescence through enzyme activity of β-galactosidase. A very significant increase of senescent blue cells was observed after NUPR1 depletion with specific siRNA (B), and with 10 µM of Compound-15 treatment for two days (C). Surface recovery was measured after 48 h with or without 10 µM of Compound-15.

Figure S1

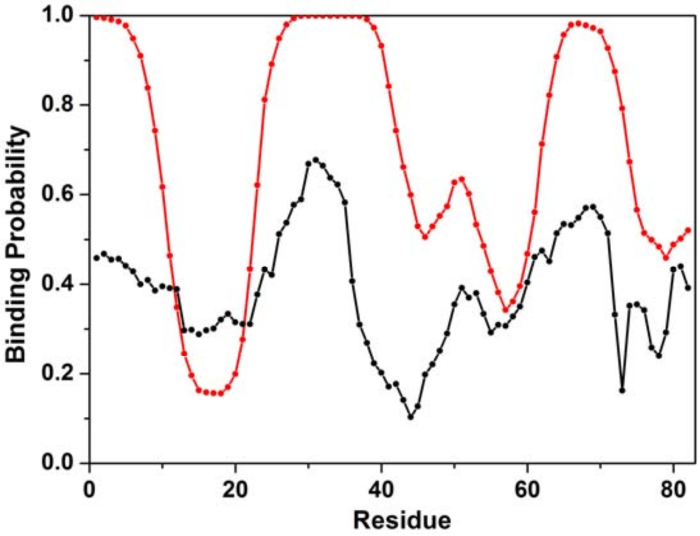

Figure S2

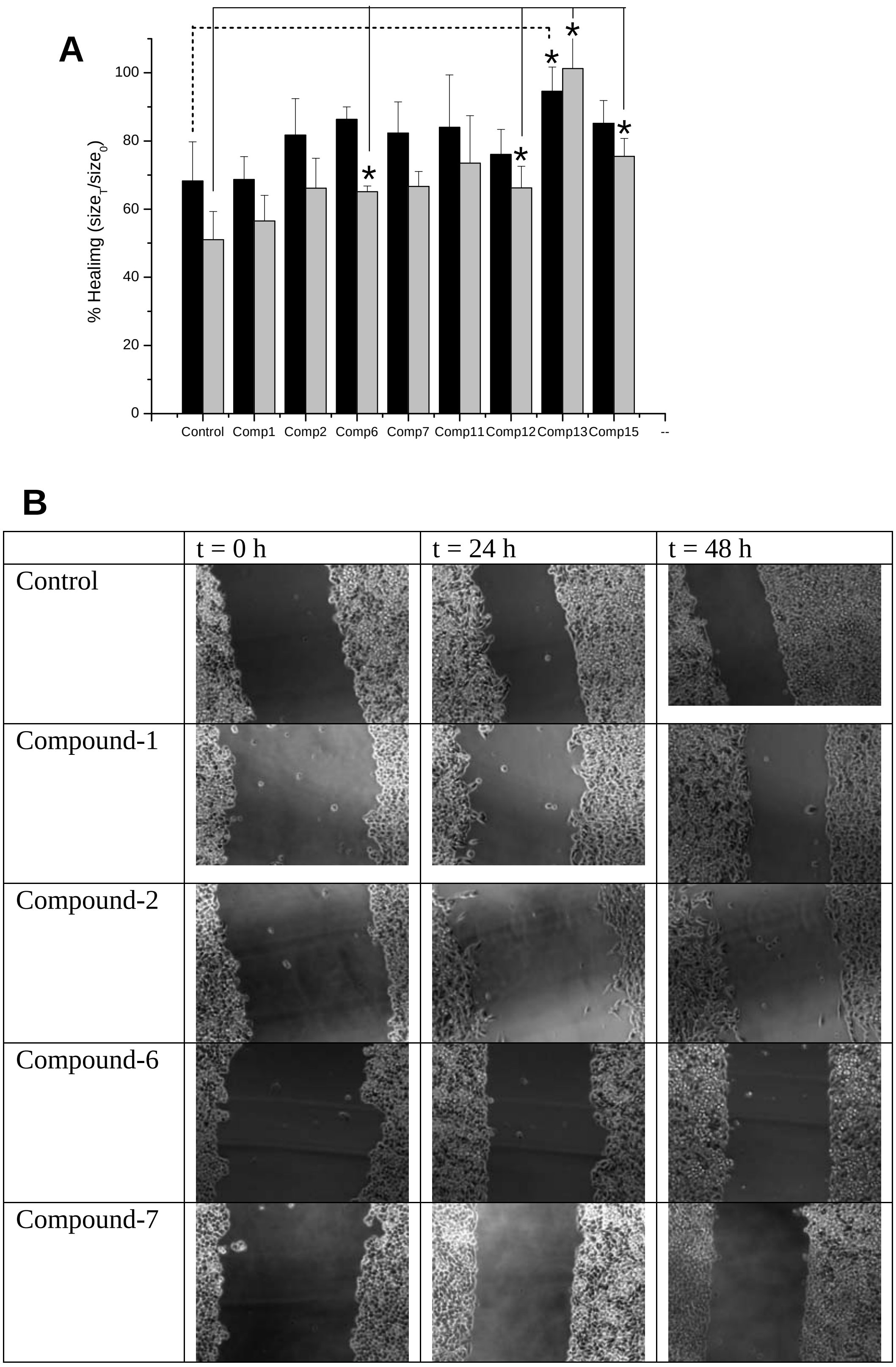
A
100
80
60
40
20
0
% Healimg (sizeT/size0)
Control Comp1 Comp2 Comp6 Comp7 Comp11 Comp12 Comp13 Comp15 --
B
t = 0 h
t = 24 h
t = 48 h
Control
Compound-1
Compound-2
Compound-6
Compound-7

Compound-11	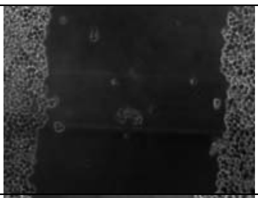	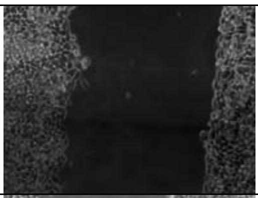	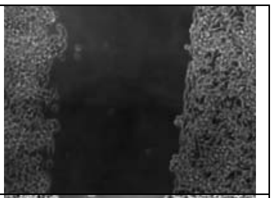
Compound-12	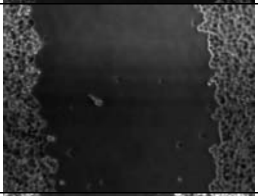	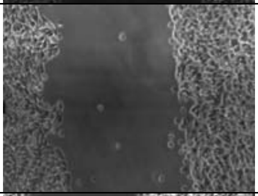	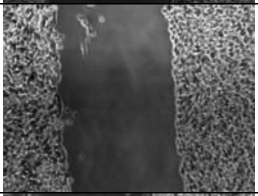
Compound-13	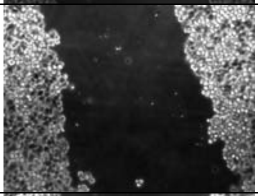	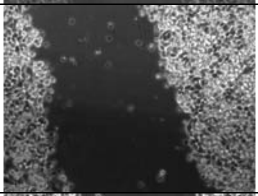	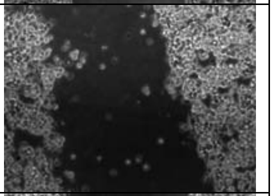
Compound-15	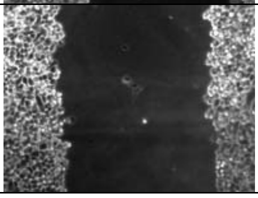	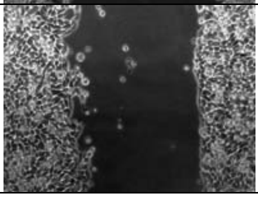	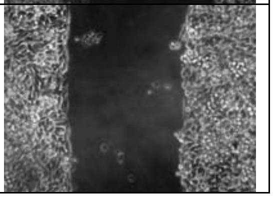

Figure S3

A

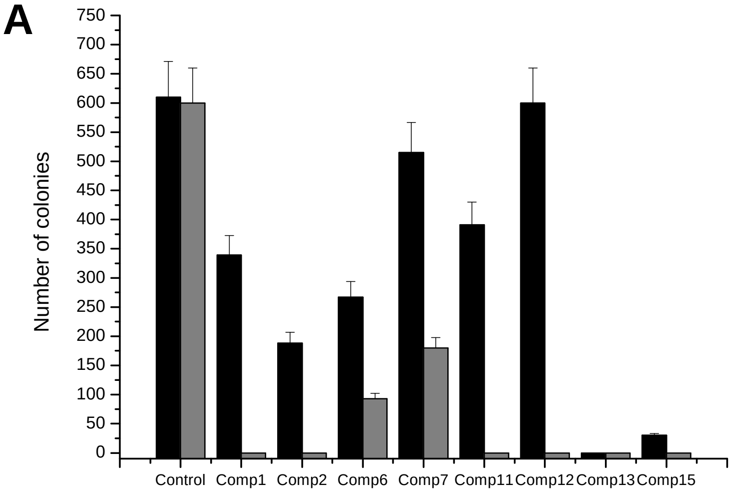

B

	10 µM compound	50 µM compound
Control	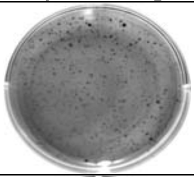	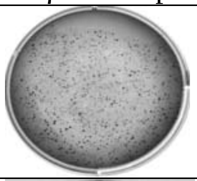
Compound-1	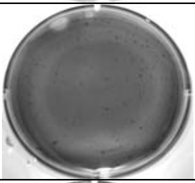	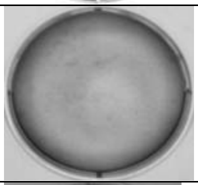
Compound-2	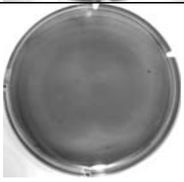	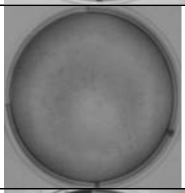
Compound-6	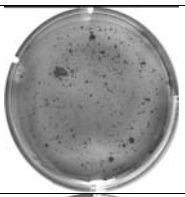	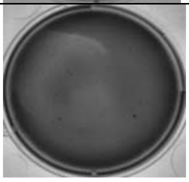
Compound-7	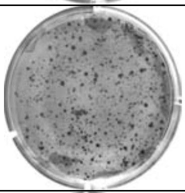	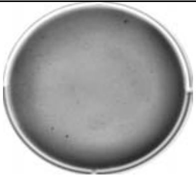
Compound-11	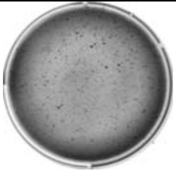	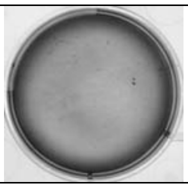

Compound-12	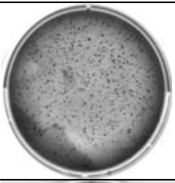	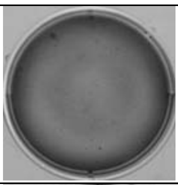
Compound-13	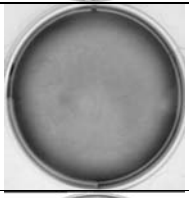	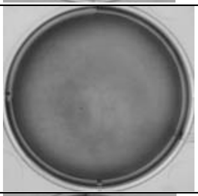
Compound-15	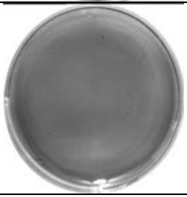	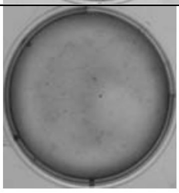

Figure S4

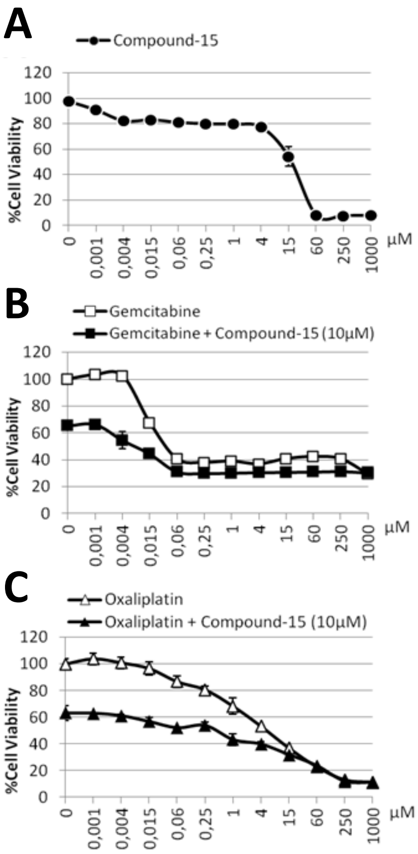
A
Compound-15
%Cell Viability
120
100
80
60
40
20
0
0
0,001
0,004
0,015
0,06
0,25
1
4
15
60
250
1000
µM
B
Gemcitabine
Gemcitabine + Compound-15 (10µM)
%Cell Viability
C
Oxaliplatin
Oxaliplatin + Compound-15 (10µM)
%Cell Viability

Figure S5

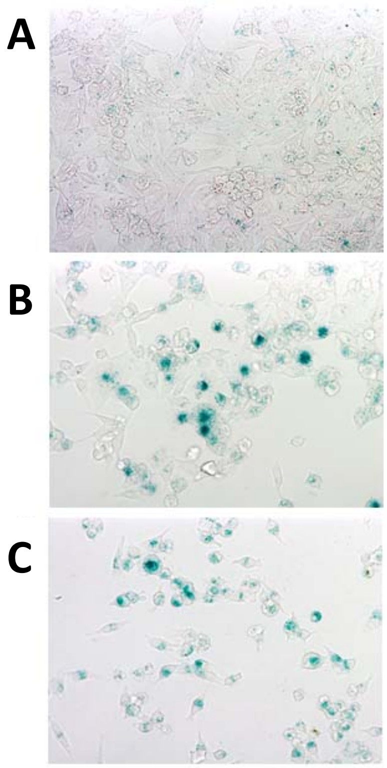

Supplement: Supplementary Information [file srep39732-s1.pdf]
